# A Microfluidic System of Gene Transfer by Ultrasound

**DOI:** 10.3390/mi13071126

**Published:** 2022-07-16

**Authors:** Cuimin Sun, Menghua Zhang, Guangyong Huang, Ping Zhang, Ronghui Lin, Xiangjun Wang, Hui You

**Affiliations:** 1 School of Computer, Electronics and Information, Guangxi University, Nanning 530004, China; cmsun@gxu.edu.cn (C.S.); mhzhang@163.com (M.Z.); 2 Guangxi Colleges and Universities Key Laboratory of Multimedia Communications and Information Processing, Nanning 530004, China; 3 Department of Mechanical Engineering, Guangxi University, Nanning 530004, China; gyh@163.com; 4 Institute of Intelligent Machines, Chinese Academy of Sciences, Hefei 230031, China; pingzh@hotmail.com (P.Z.); rhlin@sina.com (R.L.); jxwang@139.com (X.W.)

**Keywords:** cell delivery, ultrasound, microfluidic

## Abstract

Ultrasonic gene transfer has advantages beyond other cell transfer techniques because ultrasound does not directly act on cells, but rather pushes the gene fragments around the cells into cells through an acoustic hole effect. Most examples reported were carried out in macro volumes with conventional ultrasonic equipment. In the present study, a MEMS focused ultrasonic transducer based on piezoelectric thin film with flexible substrate was integrated with microchannels to form a microfluidic system of gene transfer. The core part of the system is a bowl-shaped curved piezoelectric film structure that functions to focus ultrasonic waves automatically. Therefore, the low input voltage and power can obtain the sound pressure exceeding the cavitation threshold in the local area of the microchannel in order to reduce the damage to cells. The feasibility of the system is demonstrated by finite element simulation and an integrated system of MEMS ultrasonic devices and microchannels are developed to successfully carry out the ultrasonic gene transfection experiments for HeLa cells. The results show that having more ultrasonic transducers leads a higher transfection rate. The system is of great significance to the development of single-cell biochip platforms for early cancer diagnosis and assessment of cancer treatment.

## 1. Introduction

Gene transfer from exogenous sources to cells is a basic bioengineering technology and a powerful tool for characterizing the structure and functions of genes [1]. Current passive methods for naked gene transfer include the liposome mediated method [2], microinjection [3], and the virus vector transfection method [4], amongst others. The passive methods for naked gene transfer have the advantages of having no external energy supply and simple equipment, but the vector, especially the virus vector, has limitations and safety defects. For example, retroviruses with viral vectors have the potential risks of replication ability, insertion mutation, and activation of oncogenes; adenoviruses have the side effects and potential risks of causing immune responses [5]. However, the application of viral vectors is greatly limited due to limited DNA loading, difficult vector assembly, low efficiency of in vivo introduction, and high costs. Although non-viral vectors such as liposomes, polymer materials, and nano gene transporters have no defects, such as viral toxicity or immunogenicity, their transmission efficiency is very low [6,7]. The active methods for naked DNA transfer include: microinjection, particle bombardment/particle gun, electroporation, and optical methods [8,9,10]. Microinjection and particle bombardment are both interventional methods; that is, the cell membrane must be perforated to introduce DNA into cells [9]. Electroporation gene transfer also requires cell membrane perforation, which uses high-voltage electric shocks (10–20 kV/cm) to transfer the DNA plasmid into cells through the cell membrane. The cell membrane can naturally self-repair because the pores can bridge automatically within a few seconds after the electric shock. Currently, electroporation is the most commonly used method due to the advantage of high transfection efficiency and suitability for large DNA plasmids, yet the operation requires a low ionic level medium and high voltage, which can lead to high cell death [11]. In addition, the highly focused laser can also be used to generate perforations of about 1 micron in diameter in the cell membrane, which is the principle of optical gene transfer [12]. The pores formed by the laser can bridge automatically within a few seconds as a result of self-repair. However, the laser beam can easily damage the cells, which greatly limits the application of this method. Optical gene transfer is particularly suitable for single-cell transfection where only one cell is processed at a time.

**Figure 1:**
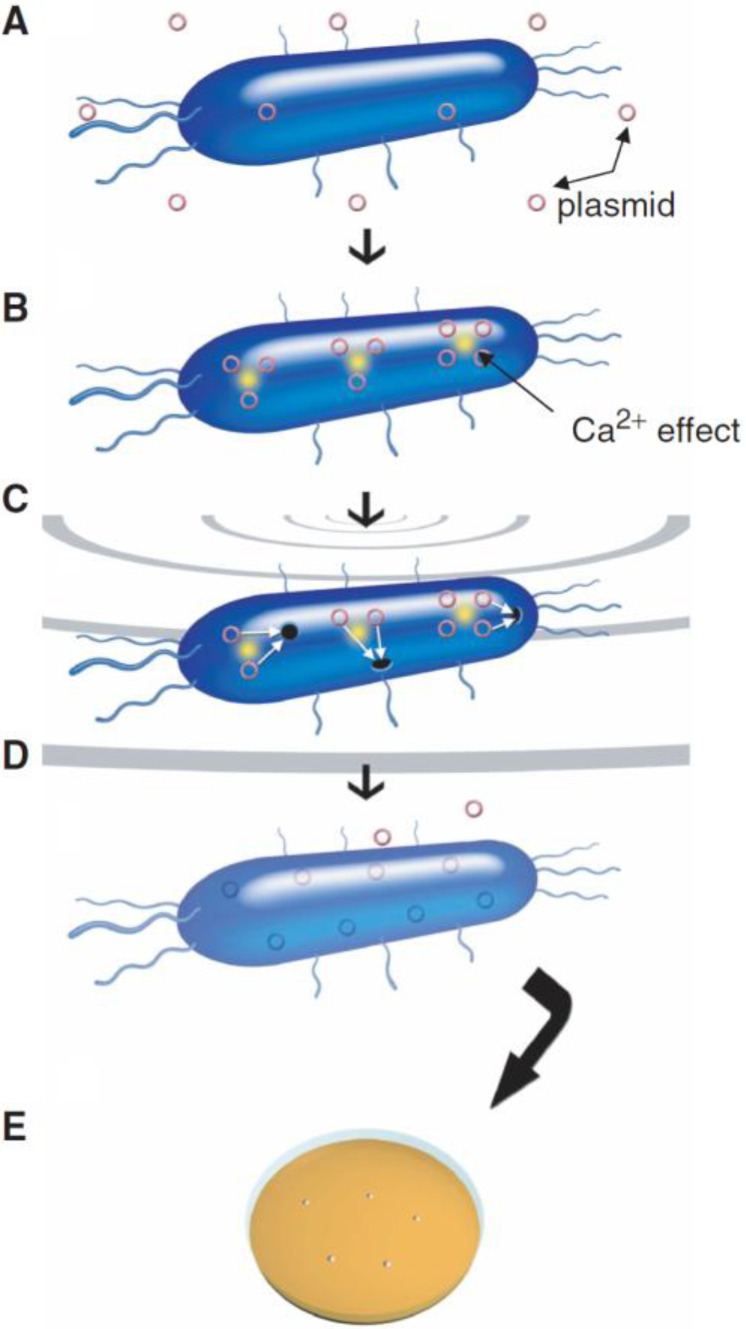
Illustration of proposed mechanism of ultrasound DNA transfer [10]. (**A**) A bacterial cell and plasmid DNA are suspended in an aqueous solution. (**B**) 50 mM CaCl_2_ promotes transformation. (**C**) Ultrasound generates pores in cell membranes through which plasmids enter the cell. (**D**) Pores are closed and plasmids are retained in the cell. (**E**) Bacteria acquire new functions after taking up plasmid DNA.

Ultrasound-mediated gene delivery, also known as sonoporation, is a recently developed cell membrane permeation technology that has been applied for DNA and drug delivery in cells [13,14,15,16]. The technology is based on the cavitation effect of ultrasonic waves. When ultrasonic waves propagate in a liquid solution, the liquid in the path will undergo alternative compression and expansion. If the ultrasonic intensity is large enough, bubbles will be formed during the compression and expansion of the liquid and they can expand to a certain extent before bursting. The period from bubble generation to burst is generally very short, usually within 1 microsecond. This process is referred to as ultrasonic cavitation [17]. The local high temperature and high pressure shock wave generated by ultrasonic cavitation can perforate a cell membrane with tiny pores with an effective diameter smaller than 100 nanometers. The pores can last for a few seconds [18], allowing larger molecules to enter the cell from the medium [19]. Under optimized conditions, the cells can survive the cavitation effect without obvious damage. Based on the self-repair mechanism, the cell membrane can bridge on its own after gene transfection [20]. The above is the general principle of ultrasound-mediated gene delivery. Figure 1 shows the mechanism of ultrasound-mediated DNA delivery and the typical steps of DNA plasmid delivery into a bacterial cell [10].

The technology is currently considered the ideal method for the transfection of DNA plasmids or fragments into cells [10,21]. It has the following advantages: (a) theoretically, DNA or RNA can be delivered to any type of cell, including bacterial cells [22], plant cells [23], and mammalian cells [24]; (b) it does not require the medium to be ion-free and can be used for cells that grow in the natural environment or the human body; (c) it is non-invasive and does not require direct contact with the cells; (d) it is easy to control the time and location of the transfer. The ultrasound can be restricted to a specific area or time period to enhance the outcome of gene transfer [25].

Existing ultrasound-mediated gene transfer technologies are all carried out under a macroscopic volume [26,27,28] using large-scale ultrasonic equipment, such as the horn-shaped ultrasonic radiator or the ultrasonic bath. Relevant studies were only carried out on a macro scale (10^5^–10^7^ cells), and resulted in averaged data. Due to the inhomogeneity of cell responses and the different life or metabolic cycles, the averaged data are often difficult to interpret. To solve this problem, it is necessary to develop new technologies and devices for single-cell operation, high-precision analysis, and high sensitivity detection. Interestingly, with microfluidic technology, the size of a biochip (lab-on-a-chip) is compatible with cell size and is suitable for single-cell operation.

Compared with macroscopic and large-volume analysis technology, the integrated biochip technology has the advantages of small reagent consumption, less residue, short reaction time, high accuracy, cost effectiveness, and disposability [29]. The use of microfluidic devices facilitates the separation, capture, location, and observation of cells. Moreover, the surface physical and chemical parameters of devices attached to the cells can be controlled, such as the local pH and temperature, in order to precisely control the local environment around the cells [30]. In the present study, we proposed an integrated system of MEMS ultrasonic devices and microchannels by enabling ultrasound-mediated cell delivery on a microfluidic scale and meeting the requirement of high-precision analysis and single-cell operation. The system is of great significance to the development of single-cell biochip platforms for early cancer diagnosis and assessment of cancer treatment.

## 2. Materials and Methods

### 2.1. Microfluidic Ultrasound-Medicated Cell Delivery System Design

**Figure 2:**
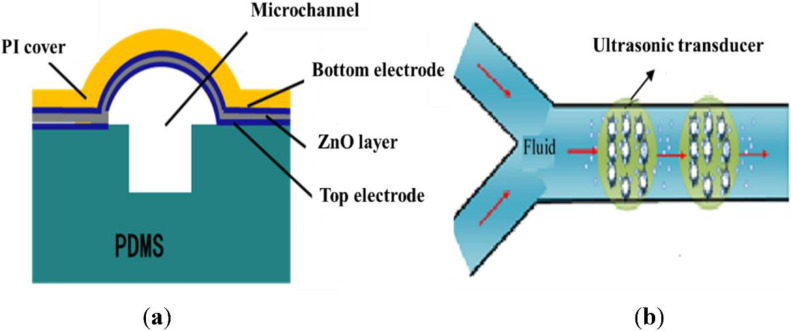
Schematic diagram of the microfluidic ultrasound gene transfer system MEMS (**a**) self-focusing ultrasonic transducer; (**b**) microchannel.

The microchannel was assembled to the transducer array to form the microfluidic ultrasound-medicated delivery system, as shown in Figure 2. The core components of the system included the MEMS piezoelectric ultrasonic transducers and the microchannels. The microchannel was etched from silicon wafers, and the cover was made of polyimide (PI). The piezoelectric ultrasonic transducer was located inside the PI cover and was composed of a piezoelectric film deposited on a spherical structure. The spherical shape could produce an excellent ultrasonic focusing effect. By applying an excitation voltage on the piezoelectric film, a slight vibration was generated. The generated ultrasonic waves entered the microchannel and acted on the flowing fluid in the tubing. The microchannel was connected to a syringe pump, which provided the flow to carry the cells and DNA plasmids through the area in the focus of the ultrasound. If the excitation voltage is not too high and the intensity and frequency of the ultrasonic wave are appropriate, microbubbles are generated in the fluid, i.e., cavitation. The cells in the area were subject to the shock wave caused by the cavitation and the localized high temperature and high pressure, and tiny pores were generated in the cell membrane, through which DNA could be delivered to the cell. After the cell exited the ultrasonic irradiation area, the tiny pores in the cell membrane bridged automatically in a short time, and the DNA was kept inside. At this point, the ultrasonic DNA transfer was completed.

### 2.2. Simulation

Although the focus of a spherical ultrasonic source has been a topic of fundamental interest for many years, several of its aspects, such as non-linear performance, are still not fully understood [31]. As there is no analytical solution for a spherical focusing field, some approximation models have been derived [32,33,34]. For continuous waves, under the assumptions of source piston movement and plane wave propagation, B.G.Lucas derived an integral model. However, the model is obtained by converting the Helmholtz equation into a parabola equation with the restriction of Fresnel approximation, and is only applicable to shallow curved spherical sources with a feature size much larger than the wavelength of the sound generated [31]. The MEMS ultrasonic transducer we propose is heavily curved to achieve a strong focusing effect, and its feature size is similar to the ultrasound wavelength; therefore, we cannot use Lucas’ model. the system consists of several types of materials and interfaces, which renders it too complex for an analytical solution. In addition, the interaction between the thin-film piezoelectric material and the acoustic media cannot be omitted, as is the case in the analytical approach. To obtain an accurate solution, Finite Element Analysis(FEA)based piezo-acoustic coupling Catalysis is required. COMSOL Multiphysics^®^ (version 5.2, COMSOL, Nanning, China)in combination with its acoustics and MEMS modules provides the means to perform this simulation.

**Figure 3:**
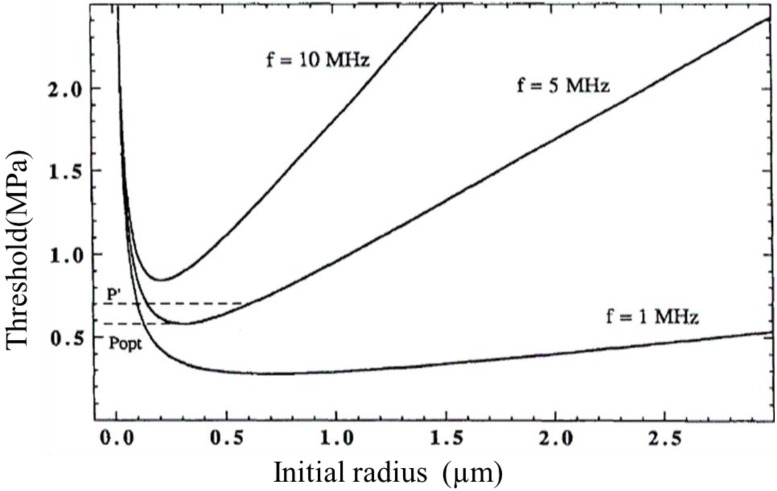
Plot of the cavitation threshold in water as a function of initial nucleus radius for three frequencies ultrasound: 1, 5, and 10 MHz [35].

According to bubble dynamics, there is a minimum acoustic pressure amplitude required to cause the cavitation, which is defined as the cavitation threshold. It is dependent on many facts including liquid properties such as surface tension, viscosity, inertia, the frequency of the ultrasound used, the initial nucleus radius, etc. According to an analytical model [35], the cavitation threshold increases with decreasing nucleus size due to surface tension, and increases with increasing nucleus size due to inertial and viscous effects. Figure 3 shows the cavitation threshold in water as a function of initial nucleus radius for three frequencies of ultrasound: 1, 5, and 10 MHz [35].

According to the analytical model, there is an optimal bubble size at a given frequency for which the threshold is a minimum. This effect is shown in the computed threshold curves in Figure 3. At higher frequencies, the optimal bubble size for cavitation nucleation decreases because inertial and viscous effects increase with frequency. The minimum pressure threshold from the theory of this optimal bubble size has been computed [36]. Table 1 shows the results for the ultrasound frequency between 1 to 5 MHz.

**Figure 4:**
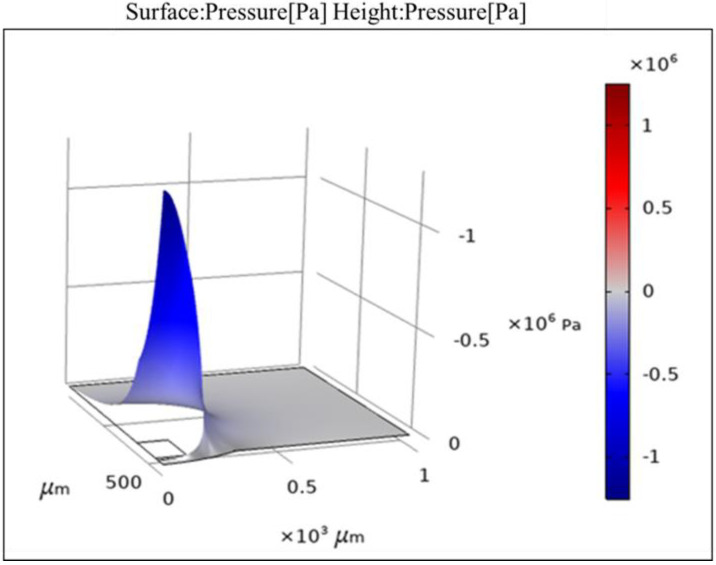
The whole field generated by a device with PI cover for an actuation frequency of 1 MHz and an actuation voltage of 100 V AC.

**Figure 5:**
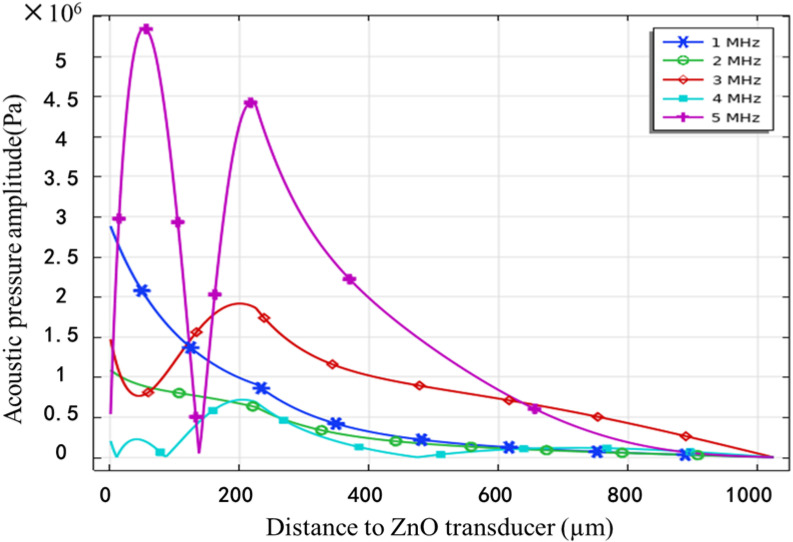
Acoustic pressure distribution along in the normal axis, generated by the device with the cover.

The application-case of the proposed device that the minimum bubble size is to be collapsed is 1 μm, hence the maximum frequency required in operation is 6 MHz. The piezo-acoustic coupling modelling of the system has been carried out with an actuation voltage of 100 V AC and actuation frequencies between 1 MHz and 5 MHz. Figure 4 shows the acoustic pressure distribution in the entire field when an actuation voltage of 100 V AC at 1 MHz is applied. Figure 5 shows a comparison of the acoustic pressure in the liquid along the normal axis for actuation frequencies between 1 MHz and 5 MHz and an actuation voltage of 100 V AC.

Comparing Figure 5 and Table 1, it can be found that when the AC excitation voltage was 100 volts, the maximum ultrasonic pressure of the fluid in the microchannel was higher than the minimum cavitation threshold of water at the frequencies of 1, 2, and 5 MHz; while at 3 and 4 MHz, the maximum ultrasonic pressure was slightly lower than the minimum cavitation threshold of the corresponding frequency. Furthermore, when the AC excitation voltage increased to 120 V and 180 V at 3 and 4 MHz respectively, the maximum ultrasonic pressure in the microchannel exceeded the minimum cavitation threshold of water at the corresponding frequency. The findings suggested that it was possible to generate microbubbles and form cavitation when the excitation voltage was not too high. Comparing Figure 3 and Figure 5, it can be seen that when the excitation frequency was 1–5 MHz, cavitation with a bubble diameter of 1–5 μm was formed in the microchannel. The simulation results showed that the system proposed in this study was able to form ultrasonic cavitation in the microfluid theoretically.

## 3. Results

### 3.1. Spherical Self-Focusing MEMS Ultrasonic Transducer

**Figure 6:**
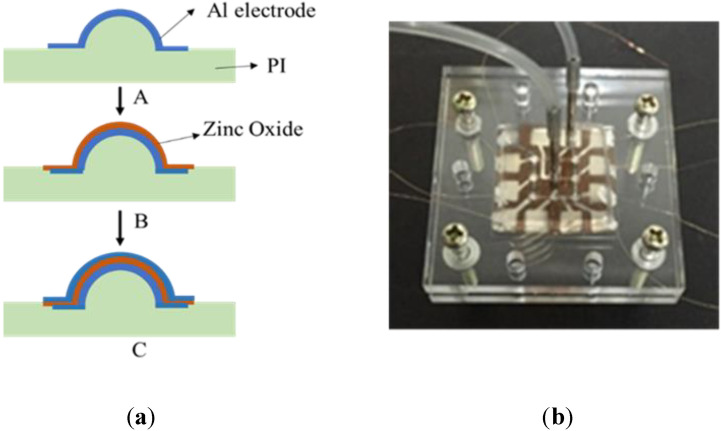
(**a**) Flow chart of preparation of the self-focusing ultrasonic transducer array on a PI substrate; (**b**) a picture of the microfluidic transfection biochip.

The spherical self-focusing MEMS ultrasonic transducer was composed of a curved piezoelectric film and a bowl-shaped structure for ultrasonic focusing. ZnO was used as the material of the bowl-shaped piezoelectric film. The manufacturing process included soft imprinting and magnetron sputtering coating. PI was used for the microchannel cover and the spherical cap. Polyimide solution was poured into a microstructure mold. After drying, curing, and demolding, a solid (flexible) replica of the microstructure was obtained. This technique was used to make the cover of the microchannel and the spherical cap. The mold was made from the embossing of a high-precision steel ball. With the above technique, the high-precision steel ball and PDMS soft imprinting were used to make the spherical MEMS microstructure array on the PI substrate, and ZnO piezoelectric film with a thickness of 4 microns was sputtered, including the upper and lower electrodes. The specific steps are shown in Figure 6a. After obtaining the spherical structure, the PI was put in the magnetron sputtering apparatus and a stainless-steel plate was used as a baffle to define the shape of the lower electrode. The A1 electrode was prepared using DC magnetron sputtering. The final thickness of the lower electrode was ~500 nanometers. After the lower electrode was deposited, the zinc oxide was produced by RF magnetron sputtering. Similarly, another stainless-steel baffle was used to define the shape. The thickness of the deposited zinc oxide was 4 microns. The Al upper electrode was prepared by DC magnetron sputtering, and a stainless steel baffle was used to define the shape.

SU-8 photoresist (negative photoresist) with a thickness of 100 microns was used. Through ultraviolet exposure and development, a convex microchannel mold was made. PDMS was poured into the mold. After curing and demolding, a PDMS microchannel with a height of 100 microns and a width of 200 microns was obtained. The ultrasonic transducer was assembled to the microchannel with screws. The screws generated a compressive pressure between the two surfaces. Due to the large elasticity of PDMS, it deformed under the compression and sealed the microchannel. One advantage of the screws is simple assembly and disassembly. Since the microchannel and the transducer were not chemically bonded together, it was very convenient to disassemble and replace them if necessary. Figure 6b is the successfully prepared microfluidic gene transfection biochip.

### 3.2. Performance Test of the Ultrasonic Transducer Array

**Figure 7:**
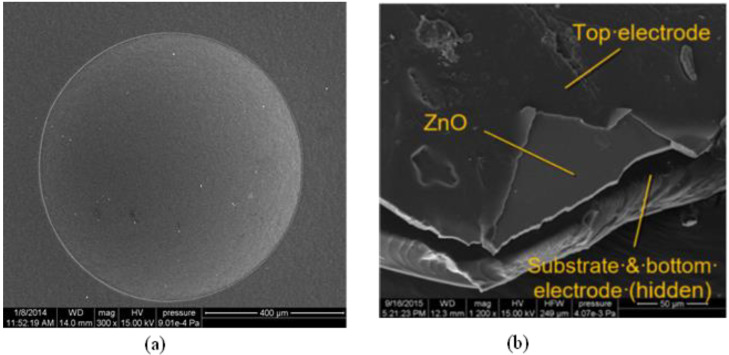
SEM images of the ZnO piezoelectric film self-focusing ultrasonic transducer (**a**) spherical cap and (**b**) ZnO film and electrode.

Surface roughness is a critical factor for the performance of MEMS ultrasonic transducers. If the roughness is too high, the deposition of the upper electrode and the ultrasonic wave emission can be affected. The prepared transducer was observed under a scanning electron microscope (SEM). It can be observed that the convex spherical structure was a standard circle (Figure 7a); the ZnO film and the upper electrode were also observed (Figure 7b). Based on the SEM images, it can be observed that the ZnO film had good quality, and the thickness of the piezoelectric film reached 4 microns. The surface roughness of the film was low, and it adhered well to the substrate.

**Figure 8:**
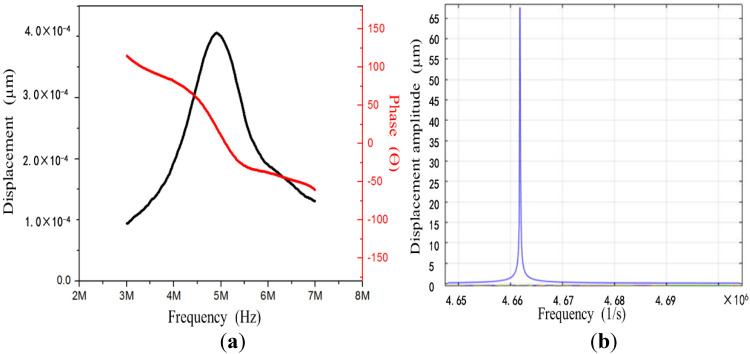
(**a**) Frequency response curve of the ultrasonic transducer array (laser Doppler vibrometer); (**b**) vibration modes and frequency response of the ultrasonic transducer array (finite element simulation).

The frequency response of the ultrasonic transducer array was tested with a laser Doppler vibrometer (Displacement Unit., MLD-821, NEOARK Corp, Tokyo, Japan), and the result is shown in Figure 8a. It can be observed that the transducer had the most significant displacement at 5 MHz. The phase angle was 0, which indicates that its resonant frequency was around 5 MHz. Moreover, a finite element simulation of the transducer was carried out, which showed that the resonant frequency was about 4.66 MHz (Figure 8b). This was consistent with the result of the laser Doppler vibrometer. The resonant frequency met the design requirements and could produce ultrasonic cavitation in a microfluid.

**Figure 9:**
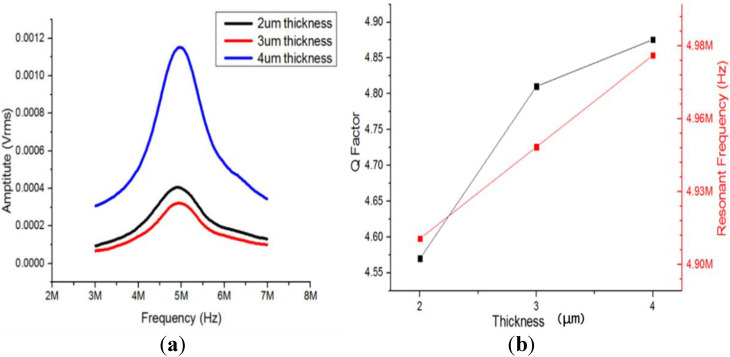
(**a**) Influence of the thickness of the ZnO piezoelectric film on the Q value of the transducer; (**b**) influence of the thickness of the ZnO piezoelectric film on the resonant frequency of the transducer.

In addition, we conducted experiments and numerical simulations on the factors of the performance of the ultrasonic transducer. The results showed that the shape of the transducer had a great impact on performance parameters such as the resonant frequency and Q value. The effect of the thickness of the ZnO piezoelectric film on the performance of the transducer was studied. By controlling the magnetron sputtering parameters, ZnO piezoelectric films of different thicknesses (2–4 microns) were prepared, and the corresponding frequency response was tested. The results are shown in Figure 9. It can be seen that: (1) with the increase of the thickness of the ZnO piezoelectric film, the resonant frequency of the transducer increased slightly; (2) with the increase of the thickness, the piezoelectric effect was more significant and the crystal orientation and uniformity were better, leading to an increase in the Q value. Thus, it can be predicted that if the film thickness was further increased to 10 microns or higher, the performance of the transducer would be significantly improved.

### 3.3. Cavitation Effect

The sonochemical method refers to the promotion of chemical reactions through ultrasonic cavitation and the reflection of the generation and intensity of the ultrasonic cavitation effect through sonochemical products [37]. An advantage of the method is that it is not affected by the environment. Once the ultrasonic cavitation energy exceeds the threshold required for the chemical reactions, which causes molecular bond destruction and recombination, new products are generated that can then be used to characterize the generation of ultrasonic cavitation. Based on the amount of the new products, the cavitation intensity can be quantitatively analyzed. The sonochemical method is universal, simple, economical, practical, and does not require complex equipment. In this study, the iodine release method was used.

The main principle of the iodine release method is the oxidation-reduction reaction of the KI solution under the ultrasound. The iodide ions in the solution will be oxidized into essential iodine and precipitate. By putting starch into the solution, the mixture will turn blue. Then, the amount of precipitated essential iodine is measured through the titration of the sodium thiosulfate solution. From there, the intensity of the cavitation effect can be characterized. The iodine release method is intuitive and reliable because the color change process is visible.

**Figure 10:**
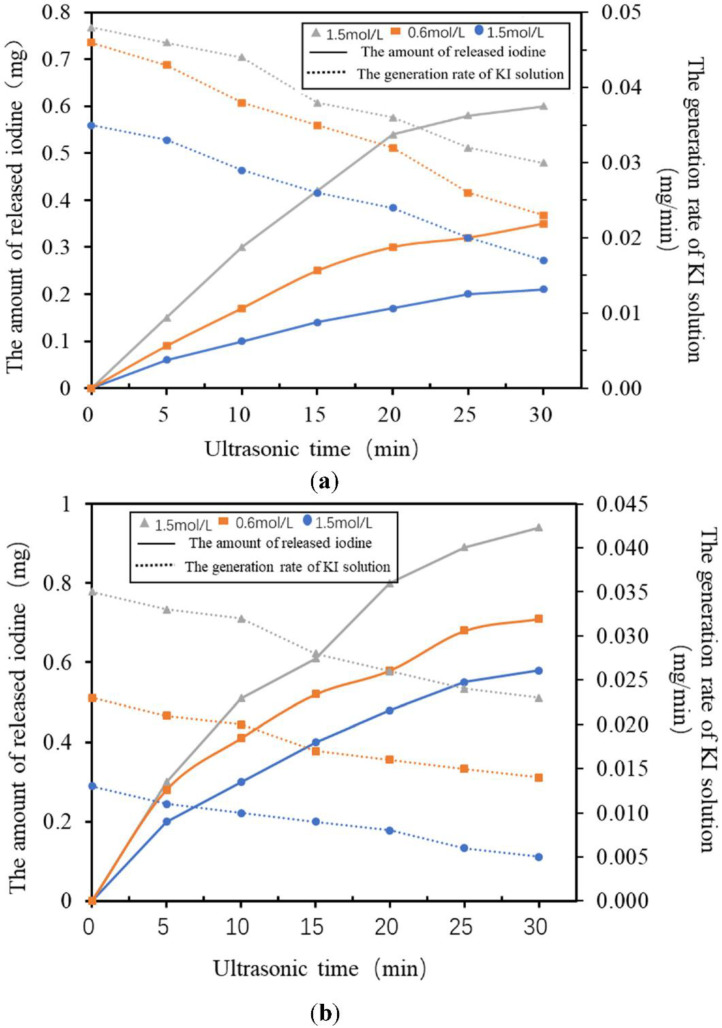
Relationship between the yield of essential iodine, the generation rate and the time of ultrasonic irradiation in KI solutions with different initial concentrations: (**a**) 2 MHz and (**b**) 3 MHz.

Figure 10 shows the cavitation yield in KI solutions with different initial concentrations. It can be seen that higher concentrations lead to larger rates of essential iodine generation. For KI solutions with the same concentration, the ultrasonic irradiation time increased and the rate of essential iodine generation decreased. This method can test the performance of the ultrasonic transducer easily. If there is a fast rate of iodine generation, it means that the transducer has a high energy conversion efficiency.

### 3.4. Ultrasonic Gene Transfection Experiment

**Figure 11:**
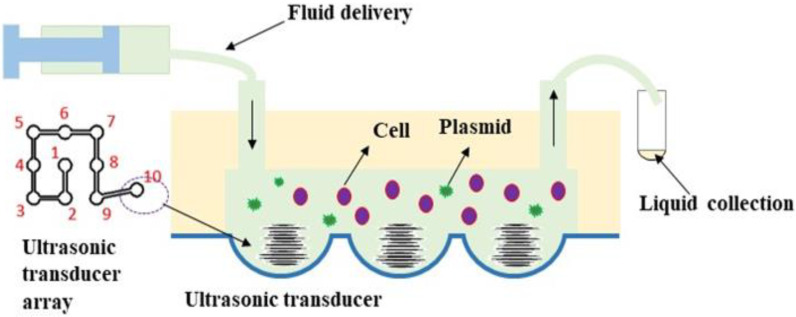
Schematic diagram of the ultrasonic gene transfection experiment.

The microfluidic system in Figure 2 was used to carry out the ultrasonic gene transfection experiments for HeLa cells. The whole experiment process is shown in Figure 11. The transducers were placed in an array at the bottom of the microchannel, in which the No. 1 transducer was the reagent inlet, No. 2–9 the ultrasonic irradiation area, and No. 10 the reagent outlet. The plasmids and cells were mixed in a certain proportion into the injection pump and sent into the microchannel at a speed of 50 nL/s. The cells would undergo multiple rounds of ultrasonic irradiation in the picture, flowing through the channel and then entering the liquid collection device. The resonant frequency of the 8 piezoelectric film ultrasonic transducers is approximately 3 MHz, and the distance between each transducer is 8 mm. One or more of the transducers can be selected to work according to the purpose of cell transduction. In the microfluidic environment, flowing cells pass through the microchannel and are subjected to the cavitation effect of ultrasonic transducer at the bottom to generate temporary micropores, through which genes and other substances can enter cells to realize gene transfection. Ultrasonic transducers generate heat when operating, which is facilitated by microfluidic conditions. In the microchannel, a small number of cells pass through the bottom transducer in turn with different flow rates, and the time of ultrasonic cavitation of cells is also different, resulting in different transduction effects. In the microfluidic environment, the transducers are arranged in array, and the cells can be subjected to the ultrasonic action of multiple transducers, which is more conducive to gene introduction.

The human renal epithelial cell line (293T cell) was used to prepare single cell suspension, and the cells were irradiated with the No. 2 ultrasonic transducer. The effect of ultrasound on cell membrane was evaluated by double staining with the fluorescein diabetic acid (FDA) and propidium iodide (PI). The PI can penetrate the membrane of damaged cells and emit red fluorescent light after combining with intracellular DNA. Moreover, active cells emit green fluorescent light with the FDA.

**Figure 12:**
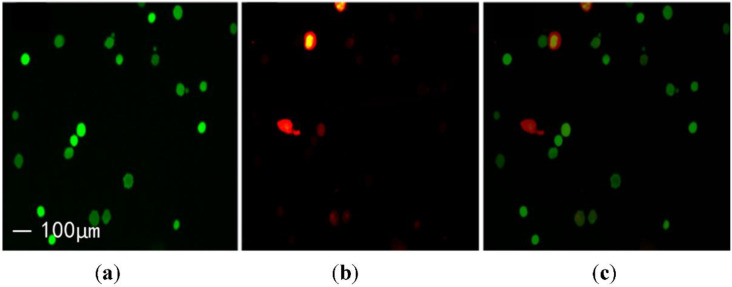
Results of 293T cell stained by FDA-PI: (**a**) live cells stained by FDA; (**b**) cells with damaged membranes stained by PI; (**c**) superposition of Figures (**a**,**b**).

As shown in Figure 12, cells only emitting green fluorescence indicate that their cell membranes may not be perforated, while cells only emitting red fluorescence may fail to maintain the integrity of cell membranes due to excessive ultrasonic irradiation, resulting in cell death. If a certain cell emits green and red fluorescent lights after FDA and PI staining, it means that the cell is perforated successfully by ultrasound and the membrane integrity is repair.

HeLa cells were transfected with green fluorescent protein granules (PGFP-N2), and the cell activity was detected by MTT assay. 1.6μg plasmid was added to group A, group B, group C, and group D. No ultrasonic transducer was turned on in group A, ultrasonic transducer No. 2 was turned on in group B for 20 s, ultrasonic transducer No. 4 was turned on in group C for 20 s, and ultrasonic transducers No. 2 and 4 were turned on in group D for 20 s. The cells in each group were placed under a fluorescence microscope to observe the expression of green fluorescent protein granules (PGFP-N2). The collected solution was cultured in a 12 well plate at 37 °C in a 5% carbon dioxide incubator. After 6 h, the fresh medium containing fetal bovine serum and double antibodies was replaced and continued to culture for 24–48 h. The cells in each group were randomly selected from 6 visual fields under a high-power microscope and the cells expressing green fluorescent protein in each visual field were counted. Each group counted 3 holes. Transfection efficiency = cells with green fluorescence/total cells × 100%.

**Figure 13:**
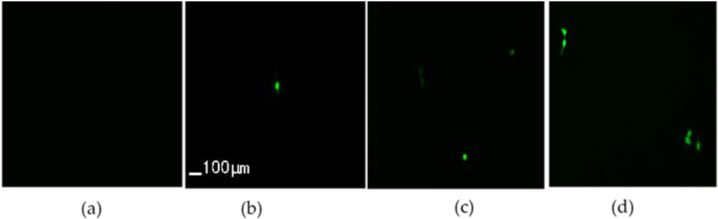
Results of green fluorescent protein expression in HeLa cells (×200): (**a**) only plasmids added; (**b**) plasmids added and sonicated with No.2 transducer for 20 s; (**c**) plasmids added and sonicated with No.4 transducer for 20 s; (**d**) plasmids added and sonicated with No.2 and 4 transducers simultaneously for 20 s.

Under a fluorescence microscope, the gene import effect is shown in Figure 13 and the green cells indicate successful import. Figure 13a shows the control group with plasmids added to the cell suspension without ultrasound. Figure 13b,c respectively represent the results of acting with one transducer, and Figure 13d represents the results of acting with two transducers simultaneously.

The transfection efficiency was improved with both transducers acting. It can be speculated that if more transducers act simultaneously, their import efficiency will be further improved. This suggests that cell import of MEMS ultrasound array is a potential technology because MEMS transducers have the potential to make large-scale arrays, which can greatly increase the import efficiency when multiple transducers act together.

## 4. Discussion

This study proposed an integrated system of a MEMS ultrasonic device and microchannel that enabled ultrasound-mediated gene delivery on a microfluidic scale, thus meeting the needs of high-precision analysis and single-cell analysis. The combination of microfluidic technology and MEMS technology in gene delivery is an innovation point of this study. The developed transfection device is portable and the medium is transparent, which facilitates observation under the microscope. The use of a syringe pump can achieve automated transfection. In the microfluidic system, the microfluidic tubing was equipped with multiple ultrasonic transducers with different frequencies, thereby adjusting the cavitation threshold or increasing gene transduction efficiency by adding an ultrasonic contrast agent. Using the proposed method, we successfully achieved gene transfection into HeLa cells. However, the current transfection effect is not ideal. Improvements to the system are needed, such as the duration of ultrasound irradiation. In summary, the microfluidic ultrasound-medicated cell delivery system accomplished gene transfection in a microfluidic environment and laid a foundation for its application in targeted cells or tissue-specific transduction. If the proposed method is combined with cell capture technology, it is expected that single-cell specific-gene transduction can be accomplished, which will be of great significance for the development of single-cell gene transfection devices.

## 5. Patents

The work reported in this manuscript has applied for the invention patent “microfluidic system for single cell ultrasonic gene delivery and its delivery method” in China, and the patent number is CN105567562a.

## Figures and Tables

**Table 1:** Minimum cavitation threshold Popt at different ultrasound frequency f.

f (MHz)	Popt (MPa)
1	0.262
2	0.364
3	0.442
4	0.507
5	0.564
